# *SLC30A9* mutation affecting intracellular zinc homeostasis causes a novel cerebro-renal syndrome

**DOI:** 10.1093/brain/awx013

**Published:** 2017-02-09

**Authors:** Yonatan Perez, Zamir Shorer, Keren Liani-Leibson, Pauline Chabosseau, Rotem Kadir, Michael Volodarsky, Daniel Halperin, Shiran Barber-Zucker, Hanna Shalev, Ruth Schreiber, Libe Gradstein, Evgenia Gurevich, Raz Zarivach, Guy A. Rutter, Daniel Landau, Ohad S. Birk

**Affiliations:** 1 The Morris Kahn Laboratory of Human Genetics, National Institute for Biotechnology in the Negev and Faculty of Health Sciences, Ben Gurion University of the Negev, Beer Sheva 84105, Israel; 2 Pediatric Neurology unit, Division of Pediatrics, Soroka University Medical Center, Faculty of Health Sciences, Ben Gurion University of the Negev, Beer Sheva 84101, Israel; 3 Section of Cell Biology and Functional Genomics, Division of Diabetes Endocrinology and Metabolism, Department of Medicine, Imperial College London, London W12 0NN, UK; 4 Department of Life Sciences and the National Institute for Biotechnology in the Negev, Ben-Gurion University of the Negev, Beer Sheva, 8410501, Israel; 5 Pediatric Nephology unit, Division of Pediatrics, Soroka University Medical Center, Faculty of Health Sciences, Ben Gurion University of the Negev, Beer Sheva 84101, Israel; 6 Department of Ophthalmolgy, Soroka Medical Center and Clalit Health Services, Faculty of Health Sciences, Ben Gurion University of the Negev, Beer-Sheva, BeerSheva 84105, Israel; 7 Genetics Institute, Soroka University Medical Center, Ben Gurion University of the Negev, Beer Sheva 84101, Israel

**Keywords:** SLC30A9, ZnT9, Cerebro-renal syndrome, neurodevelopmental regression, zinc homeostasis

## Abstract

A novel autosomal recessive cerebro-renal syndrome was identified in consanguineous Bedouin kindred: neurological deterioration was evident as of early age, progressing into severe intellectual disability, profound ataxia, camptocormia and oculomotor apraxia. Brain MRI was normal. Four of the six affected individuals also had early-onset nephropathy with features of tubulo-interstitial nephritis, hypertension and tendency for hyperkalemia, though none had rapid deterioration of renal function. Genome wide linkage analysis identified an ∼18 Mb disease-associated locus on chromosome 4 (maximal logarithm of odds score 4.4 at D4S2971; θ = 0). Whole exome sequencing identified a single mutation in *SLC30A9* within this locus, segregating as expected within the kindred and not found in a homozygous state in 300 Bedouin controls. We showed that *SLC30A9* (solute carrier family 30 member 9; also known as ZnT-9) is ubiquitously expressed with high levels in cerebellum, skeletal muscle, thymus and kidney. Confocal analysis of SH-SY5Y cells overexpressing *SLC30A9* fused to enhanced green fluorescent protein demonstrated vesicular cytosolic localization associated with the endoplasmic reticulum, not co-localizing with endosomal or Golgi markers. *SLC30A9* encodes a putative zinc transporter (by similarity) previously associated with Wnt signalling. However, using dual-luciferase reporter assay in SH-SY5Y cells we showed that Wnt signalling was not affected by the mutation. Based on protein modelling, the identified mutation is expected to affect SLC30A9’s highly conserved cation efflux domain, putatively disrupting its transmembrane helix structure. Cytosolic Zn^2+^ measurements in HEK293 cells overexpressing wild-type and mutant *SLC30A9* showed lower zinc concentration within mutant rather than wild-type *SLC30A9* cells. This suggests that SLC30A9 has zinc transport properties affecting intracellular zinc homeostasis, and that the molecular mechanism of the disease is through defective function of this novel activity of *SLC30A9* rather than by a defect in its previously described role in transcriptional activation of Wnt signalling.

## Teaser

Few cerebro-renal syndromes have been described to date. Perez *et al.* identify a novel autosomal recessive cerebro-renal syndrome in a consanguineous Bedouin kindred, caused by a mutation in *SLC30A9.* The mutation disrupts the role of SLC30A9 in Zn^2+^ transport, leading to impaired regulation of cytosolic zinc homeostasis.

## Introduction

Cerebro-renal syndromes have mostly been defined based on the co-existence of other organs involved, such as the eyes in Lowe syndrome (Bökenkamp and Ludwig, 2016), muscles in mitochondrial cytopathies (Hall *et al.*, 2008), liver in Zellweger syndrome, etc. In some cases the type of renal involvement may aid in diagnosis, such as the unique tubulopathy in epilepsy ataxia sensorineural-deafness tubulopathy (EAST) syndrome (Bockenhauer *et al.*, 2009), or glomerulosclerosis in action myoclonus renal failure syndrome (Badhwar *et al.*, 2004). Nephronophthisis (NPHP), the most common genetic cause of end-stage renal failure in childhood and adolescence, represents a group of overlapping disorders with shared ciliopathy-related phenotypes. NPHP has been shown to be caused by mostly recessive mutations in any of at least 15 genes, most of which are associated with primary cilia. It manifests as a chronic tubulointerstitial nephropathy with accompanying renal cysts in only some cases, and may appear in combination with other phenotypes such as retinitis pigmentosa, situs inversus, liver fibrosis, cardiac malformations, and multiple developmental and neurologic abnormalities (Wolf, 2015).

**Figure 1:**
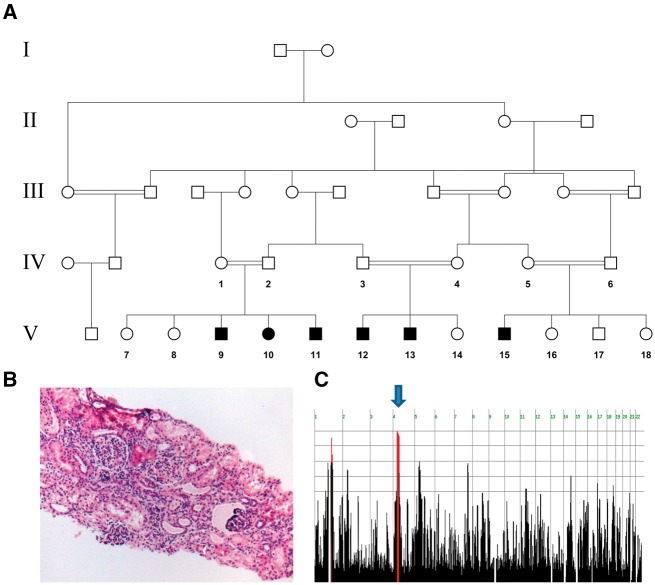
**Pedigree of studied kindred and linkage analysis.** (**A**) Pedigree of the large consanguineous Bedouin kindred studied. (**B**) Histological haematoxylin and eosin stain of renal biopsy derived from Patient V-15 at age 13 months revealed chronic tubulo-interstitial nephritis with no evident cysts or tubular dilatation. (**C**) HomozygosityMapper plot, blue arrow presenting a homozygous locus shared by all affected individuals.

Six individuals of three closely related consanguineous families of Bedouin origin (Fig. 1A) presented with an apparently autosomal recessive syndrome of early onset intellectual disability and tubulointerstitial nephropathy. All affected individuals were born at term, following uneventful pregnancies and normal birth weights, reaching normal neurodevelopmental milestones by the age of 1–2 years, but then started to deteriorate, with first signs of abnormality evident in speech delay and motor regression. Preliminary laboratory analysis ruled out association between this disease and known cerebro-renal diseases. Therefore, we set out to decipher the molecular basis underlying this syndrome.

## Materials and methods

### Subjects and clinical phenotyping

Six affected individuals of consanguineous Bedouin kindred were studied (Fig. 1A). DNA samples were obtained following approval of the Soroka Medical Center Internal Review Board. Clinical phenotyping was determined by an experienced team of paediatric neurologists, nephrologists, ophthalmologists and geneticists for all affected individuals, their parents and siblings.

### Homozygosity mapping

Genome-wide linkage analysis of all affected individuals (Fig. 1A) was performed using Illumina Omni Express Beadchip with >700K SNP loci per sample. Homozygosity mapping analysis was carried out using the open online software HomozygosityMapper (http://www.homozygositymapper.org/) (Seelow *et al.*, 2009). Fine mapping and haplotype reconstruction were performed for all available DNA samples using polymorphic markers as previously described (Perez *et al.*, 2015). Microsatellite markers used for haplotype reconstruction of the narrowed chromosome 4 locus were as follows: D4S1581, D4S3045, D4S3251, D4S3002, D4S3255, D4S3255, D4S2971 and D4S1583. All physical positions mentioned are according to the GRCh37/hg19 genome assembly. Multipoint logarithm of odds (LOD) score at the shared locus was calculated using Superlink-Online SNP 1.1 (http://cbl-hap.cs.technion.ac.il/superlink-snp/) (Silberstein *et al.*, 2006), assuming an autosomal recessive mode of inheritance with penetrance of 0.99 and disease mutant gene frequency of 0.01.

### Sequencing analysis

Whole-exome sequencing was performed as previously described (Perez *et al.*, 2015). Data were analysed through the use of QIAGEN’s Ingenuity® Variant Analysis^™^ software (www.qiagen.com/ingenuity) from QIAGEN Redwood City. Using their filtering cascade, we excluded variants that are observed with an allele frequency ≥1.0% of the genomes in the 1000 Genomes project, NHLBI ESP exomes (All) or the Allele Frequency Community. In addition, we excluded variants that appeared in a homozygous state in our in-house whole exome sequencing database of 150 Bedouin control samples. Furthermore, we kept variants that are predicted to have a deleterious effect upon protein coding sequences (e.g. Frameshift, in-frame indel, stop codon change, missense or predicted to disrupt splicing by MaxEnt Scan) and variants that are experimentally observed to be associated with a phenotype: pathogenic, possibly pathogenic or disease-associated according to The Human Gene Mutation Database (HGMD). Following the above filtering of the remaining variants, we selected only homozygous variants that were shared by the two probands (Patients V:9 and V:15) and that were located on chromosome 4 between the physical positions of SNPs rs4832940 and rs12502008. Validation and segregation analysis of the *SLC30A9* mutation was done via Sanger sequencing using the following primers: Forward 5’- TCGTGTTTTTGTTTTAGACATTACG -3’; Reverse 5’- CCTATTCCCACACTTGCTTTTC -3’ (249 bp amplicon). Annealing temperature used was 60°C and the extension time was set for 30 s.

### Multiple sequence alignment

Six representative *SLC30A9* orthologues were selected for multiple sequence alignment (MSA). All protein sequences were taken from the National Center for Biotechnology Information GenBank (http://www.ncbi.nlm.nih.gov). The RefSeq sequence accession numbers for *Homo sapiens*, *Macaca mulatta*, *Mus musculus*, *Bos taurus*, *Xenopus laevis* and *Danio rerio* of SLC30A9 orthologues used for the analysis are: NP_006336.3, AFI35986.1, XP_003585320.3, NP_001087162.1 and NP_001008575.1, respectively. Protein multiple sequence alignment was performed using Clustal Omega program (http://www.ebi.ac.uk/Tools/msa/clustalo/) (Sievers *et al.*, 2011).

### *SLC30A9* expression analysis

A panel of cDNA samples was prepared from total RNA derived of 21 normal human tissues (Clontech Laboratories), using Verso cDNA kit (Thermo Scientific^™^). Two sets of PCR primers were designed to amplify cDNA rather than genomic DNA of human *SLC30A9* and of glyceraldehyde 3-phosphate dehydrogenase (*GAPDH*) housekeeping gene as a control. Primers used for *SLC30A9* amplification (198 bp amplicon): Forward 5’-CATGGTCTCAGCATTCCTCA -3’; Reverse 5’-ACAACTCGCCCATGAAAATC -3’; Primers used for *GAPDH* (452 bp amplicon): Forward 5’- ACCACAGTCCATGCCATCAC -3’; Reverse 5’- TCCACCACCCTGTTGCTGT -3’. Annealing temperature used was 60°C, the extension time was set for 30 s and the PCR reaction repeats was of 35 cycles for both reactions.

### *SLC30A9* (ZnT-9) constructs

A custom gene synthesis plasmid; Puc57-Amp (GENEWIZ), with *SLC30A9* reference sequence (derived from GenBank; NM_006345.3) followed by a FLAG sequence (5’- GACTACAAAGACGATGACGACAAG-3’) was obtained. The full *SLC30A9* transcript (1707 nt) fused to the FLAG sequence was then excised from the Puc57-Amp custom plasmid using EcoRI and BamHI restriction enzymes and ligated into pCDNA^TM^ 3.1 (−) expression vector (Invitrogen). The c.1047_1049delGCA mutation was introduced by DpnI-mediated site directed mutagenesis. PCR primers used for mutagenesis: Forward 5’- ATGGGCATATTGTATTTTAGGATCATTAGTATCTGAAGGAG -3’; Reverse 5’- CTCCTTCAGATACTAATGATCCTAAAATACAATATGCCCAT -3’. Cloned pCDNA^TM^ 3.1 (‐) expression vectors were used for Wnt signalling measurements and for cytosolic zinc measurements. Inserts were then cut out from Cloned pCDNA^TM^ 3.1 (−) expression vectors with XhoI and BamHI restriction enzymes and ligated into pEGFP-C3 constructs. Cloned pCDNA^TM^ 3.1 (−) expression vectors were also PCR amplified with unique primers harbouring specific modifications to allow the transfer of wild-type and mutant inserts into pEGFP-N2 constructs (adding EcoRI and BamHI restriction sites with poly adenine for optimized restriction while abrogating the FLAG sequence and stop codon and maintaining reading frame). PCR primers used: Forward 5’- aaaaaaGAATTCGCCACCATGTTACCCG -3’; Reverse 5’- aaaaaaGGATCCggAGTATCTCCAAATCTACATGTCGA -3’. PCR products were then restricted using EcoRI and BamHI enzymes and ligated into pEGFP-N2 expression vector. Ligations were done using T4 DNA ligase (New England Biolabs). The final vector encodes an SLC30A9 protein fused to enhanced green fluorescent protein (EGFP) either at its N’-terminus (pEGFP-C3) followed by a FLAG sequence or SLC30A9 protein fused to EGFP at its C’-terminus (pEGFP-N2), both under control of the cytomegalovirus (CMV) promoter. These constructs were used for confocal analysis.

### Confocal analysis

SLC30A9 fused to EGFP expression plasmids (1 µg) were transfected into neuroblastoma cell line (SH-SY5Y) at 70% confluence (seeded on coverslips) in 12-well plates using Lipofectamine® 2000® (Thermo Fisher Scientific) according to the manufacturer’s instructions. At 24 h post-transfection, cells were washed twice with PBST (phosphate-buffered saline + 0.05% Tween 20), fixed in 4% paraformaldehyde for 20 min, permeabilized and blocked using Triton^™^ X-100 (0.5% v/v) in an antibody diluent reagent (E09-300; GBI Labs). Cells were then incubated with primary polyclonal goat anti-human EEA1 (Sc-6414; Santa Cruz Biotechnology), polyclonal rabbit anti- human Rab7 (Sc-10767; Santa Cruz Biotechnology), mouse monoclonal anti- human CD63 (CBL553; Merck Millipore) or polyclonal rabbit anti-human Calnexin (Sc-11397; Santa Cruz Biotechnology) antibodies for 1 h. Post-incubation, cells were washed twice with PBST and incubated with secondary donkey anti-goat IgG Alexa Fluor® 546 (A-11056; Invitrogen), goat anti-rabbit IgG Alexa Fluor® 546 (A-11010; Invitrogen) or donkey anti-mouse IgG Alexa Fluor® 546 (A-10036; Invitrogen) accordingly for 1 h. Cells were then washed twice with 1 × PBST and mounted using Vectashield containing DAPI (H-1200; Vector Laboratories). All antibodies were used at 1:1000 dilutions. The subcellular localization was visualized using an Olympus confocal microscope with a ×40 objective. Confocal images were recorded under identical conditions. Excitation was performed with a 488 nm (for EGFP), 405 nm (for DAPI) and 546 nm (for Alexa Fluor® 546) laser filtered accordingly.

### Wnt signalling measurements

To assess the activation of Wnt signalling we used standard TOP-Flash reporter assay. Neuroblastoma cells (SH-SY5Y) were plated in 12-well tissue plates to ∼60–70% confluence 12 h before transfection. Cells were then transfected with Lipofectamine® 2000 (Invitrogen) according to the manufacturer’s protocol with 500 ng of TOP-Flash plasmids (Millipore), 500 ng of the different FLAG-ZnT-9 constructs (wild-type, mutant or mock) and 25 ng of internal normalizer pGL4.74 (hRluc/TK) vector (Promega). The experiments were repeated with the negative control FOP-Flash (Millipore) to verify the activity of the TOP-Flash reporter system. Twelve hours post-transfection cells were harvested, lysed and the luciferase reporter gene assay was conducted (Dual-Luciferase Reporter Assay System, Promega, E1910). Each transfection was repeated three times and each transfected well was measured three times for luciferase activity. Luciferase activity was measured using TECAN infinite M200 instrument.

### SLC30A9 (ZnT-9) protein modelling

The ZnT-9 structure was modelled manually: 20 multiple sequence alignments were performed, each included hZnT-9 sequence, *Escherichia coli* YiiP sequence, *Shewanella oneidensis* MR-1 YiiP homologous sequence and 20 Cation Diffusion Facilitator (CDF) proteins’ sequences that were chosen randomly from 326 CDF sequences (extracted from Cubillas *et al.,* 2013). In each of these multiple sequence alignments, the residues of ZnT-9 that correspond to YiiP transmembrane helices were determined and manually compared in order to identify the most redundant residues in each position. ZnT-9 model was built manually using YiiP structure (PDB code: 3H90) (Lu and Fu, 2007) by exchanging YiiP residues in ZnT-9 homologous residues and rotation of the helices around their axes to create the chemically most likely conformation. Multiple sequence alignments and phylogenetic tree were performed using Clustal Omega (Sievers *et al.*, 2011); mutations and helices’ rotations have been implemented using Coot (Emsley and Cowtan, 2004) and Swiss-PdbViewer 4.1.0 (Guex and Peitsch, 1997); the structural model figure was prepared by PyMOL (The PyMOL Molecular Graphics System, v. 1.7.4 Schrödinger, LLC 2014).

### Cell transfection and zinc imaging

HEK293 cells were grown in Dulbecco’s modified Eagle medium containing 10% foetal calf serum, 2 mM l-glutamine, 100 units/ml penicillin and 100 µg/ml streptomycin at 37°C in a humidified atmosphere containing 5% CO_2_. Cells were plated on sterile coverslips in 6-well plates at 50–60% confluence, and co-transfected with 1.0 µg of eCALWYs-pShuttle plasmid DNA and with 1.0 µg of plasmid for ZnT9-WT or ZnT9-mut expression by using Lipofectamine® 2000 (Invitrogen) following the manufacturers’ instructions. Cells were then allowed to express proteins for 16 to 24 h. To assure overexpression, total mRNA was extracted from transfected cells, using TRIzol® according to the manufacturer’s instructions. RNA (2 µg) was reverse transcribed using the High-Capacity cDNA reverse transcription kit (Life Technologies) including random primers. Real-time PCR followed, using a SYBR® Green PCR master mix (Life Technologies) and specific primers for *SLC30A9* (ZnT9) (F: GGTTCCCTGTAGTCATCCAT; R: GAGCTTTGAGTTCTGTGCCT) and *HMBS* (F: GGCAATGCGGCTGCAA; R: GGGTACCCACGCGAATCAC) used as reference mRNA. Zinc (Zn^2+^) measurements were acquired as previously described (Chabosseau *et al.*, 2014). Cells were washed in Krebs-HEPES-bicarbonate (KHB) buffer (140 mM NaCl, 3.6 mM KCl, 0.5 mM NaH_2_PO_4_, 0.2 mM MgSO_4_, 1.5 mM CaCl_2_, 10 mM HEPES, 25 mM NaHCO_3_), which was warmed, bubbled with 95:5 O_2_/CO_2_, set to pH 7.4, and contained 3 mM glucose. Cells were maintained at 37°C throughout with a heating stage (MC60, LINKAM, Scientific Instruments), and KHB was perfused (1.5 to 2 ml/min) with additions as stated in Fig. 4B. Images were captured at 433 nm monochromatic excitation wavelength (Polychrome IV, Till photonics) using an Olympus IX-70 wide-field microscope with a 40×/1.35 NA oil immersion objective and a zyla sCMOS camera (Andor Technology) controlled by Micromanager software. Emitted light was split and filtered by a Dual-View beam splitter (Photometrics) equipped with a 505dcxn dichroic mirror and two emission filters (Chroma Technology, D470/24 for cerulean and D535/30 for citrine). Image analysis was performed with ImageJ software using a homemade macro and the fluorescence emission ratios were derived after subtracting background. Steady-state fluorescence intensity ratio citrine/cerulean (R) was measured, then maximum and minimum ratios were determined to calculate free Zn^2+^ concentration using the following formula: [Zn^2+^] = Kd(R_max_ − R)/(R − R_min_). The maximum ratio (R_max_) was obtained upon intracellular zinc chelation with 50 μM TPEN and the minimum ratio (R_min_) was obtain upon zinc saturation with 100 μM ZnCl_2_ in the presence of the Zn^2+^ ionophore, pyrithione (5 μM).

## Results

### Clinical characterization

Six individuals of three closely related consanguineous Bedouin families (Fig. 1A) presented with a syndrome that included different combinations of intellectual disability, muscle weakness, oculomotor apraxia and early onset nephropathy. All affected individuals were born at term with normal birth weights (2800–3350 g), following uneventful pregnancies. They reached normal neurodevelopmental milestones by the age of 1–2 years but then started to deteriorate. The first signs of abnormality included speech delay and motor regression. As patients advanced in age, developmental delay became global, losing previously acquired skills, and regressing into profound intellectual disability manifesting mainly in very low social and verbal skills. For instance, Patient V:11 (Fig. 1A) was born at term following normal pregnancy. Psychomotor development was normal until the age of 1.5 years, including normal milestones of standing, walking, social interaction and speech. At age 1.5 years neurological deterioration ensued. Deterioration became first evident in motor functions and frequent falls. Approximately at that same time initial signs of renal function deterioration appeared. By the age of 3 years, oculomotor apraxia became evident, as well as regression in verbal and expressive skills, capable of expressing few barely comprehensible words. Similar disease progression was evident in all other patients as well, although with some variability.

Profound ataxia of limbs and muscle weakness was present at various degrees in all patients. Patients had poor neck and limb control but were able to move limbs against gravity and mild resistance. Deep tendon reflexes were normal in all patients, without abnormal pyramidal signs, including clonus or Babinski. Mild dyskinesia was evident in all patients: most had mild choreoathetosis and dystonic postures of limbs. Older patients had marked axial hypotonia and had difficulties in walking, with camptocormia (‘bent trunk’) postures. Dysmorphic features were not evident. All patients had various degrees of congenital convergent or divergent strabismus. Eye examination revealed normal eye chambers in all, with no pigment deposits at fundus. Most children displayed wandering eye movements and lack of fixation. All had oculomotor apraxia and saccadic pursuit eye movements with variable degrees of bilateral ptosis.

Renal insufficiency was detected in four of the patients, and became evident in three of them before age 2 years. Hyperkalaemia, effectively treated through diet modification and potassium binding resin keyexalate therapy, was also seen early in disease manifestation, appearing before age 2 years in three of four children. Systemic hypertension was seen in all four. Renal sonography revealed in the four individuals with renal involvement (but not in the two other patients with only neurological symptoms) kidneys that were hyper-echogenic yet normal sized with no cysts. Renal biopsy in Patient V:15 at age 13 months revealed chronic tubulo-interstitial nephritis with no evident cysts or tubular dilatation (Fig. 1B). In spite of this early manifestation, similar to that described in NPHP type 2 (Haider *et al.*, 1998), renal function did not deteriorate during childhood. To date, at average age of 9.4 ± 2.1 years with the oldest patient aged 19 years, not a single patient has reached end-stage renal disease (Table 1).

**Table 1:** Patients characteristics

Patient ID	V:9	V:10	V:11	V:12	V:13	V:15	Mean ± SE/ % abnormal
Male/female	M	F	M	M	M	M	83% M
Age of onset (years) (neurologic/renal)	4 (N)	10 (R)(N)	2 (R)(N)	0.25 (R)	1.9(R)(N)	0.2 (R)	2.2 ± 0.8
eGFR at onset (ml/min/1.7 m^2^)	100	45	100	42	70	20	62.8 ± 13.4
Echogenic kidneys	−	+	+	+	−	+	66.7%
Max [K] (meq/l)	5.1	5	6.5	6.7	5.9	7	6.0 ± 0.3
Hypertension	−	+	+	+	−	+	66.7%
Last follow-up (years)	10.5	19	6	8.7	4.4	7.9	9.4 ± 2.1
Last follow-up eGFR (ml/min)	NA	15	NA	68	78	40	66.8 ± 13.8
Psychomotor retardation/regression	+	+	+	+	+	+	100%
Speech delay	+	+	+	+	+	+	100%
Ataxia of limbs	+	+	+	+	+	+	100%
Axial hypotonia	+	+	+	+	+	+	100%
Camptocormia	+	+	+	+	+	+	100%
Increased limb muscle tonus	+	+	+	+	+	+	100%
Dystonia/choreoathetosis	+	+	+	+	+	+	100%
Oculomotor apraxia	+	+	+	+	+	+	100%
Bilateral ptosis	NA	+	NA	+	+	+	66.7%
Strabismus	NA	+	NA	+	+	NA	50%
Brain MRI	NA	NA^b^	Normal	Normal	+/−^a^	Normal	16.7%

Patient IDs correspond with pedigrees in Fig. 1A.

[K] = serum potassium concentration (normal < 5.5 meq/l); eGFR = estimated glomerular filtration rate (normal > 90 ml/min/1.73 m^2^); + = positive; − negative; NA = not available.

^a^Periventricular white matter changes along occipital horns.

^b^Normal head CT at age 15 years.

Brain MRI of four of the patients at ages 3–10 years demonstrated no specific abnormal findings or structural/migration defects and no ‘molar tooth’ sign. Blood pH, lactate, pyruvate, creatine phosphokinase and amino acids, as well as urinary organic acids were within normal limits. Screening for congenital glycosylation defects, karyotype and chromosomal microarrays were normal.

### Genetic analysis

Genome-wide homozygosity mapping, testing all affected individuals of the kindred studied (Fig. 1A), identified a single autozygous segment shared by affected individuals: a ∼18 Mb homozygous segment on chromosome 4 between SNPs rs4832940 and rs12502008 (Fig. 1B). Segregation analysis using polymorphic markers within the above locus, performed for all available DNA samples was compatible with disease association of the chromosome 4 locus (data not shown; maximum multipoint LOD score of 4.4 at D4S2971). Sanger sequencing as well as deep sequencing (>100× coverage) of *WDR19* (NPHP13, MIM: #608151) ruled out any variants in this NPHP-associated gene within the locus.

**Figure 2:**
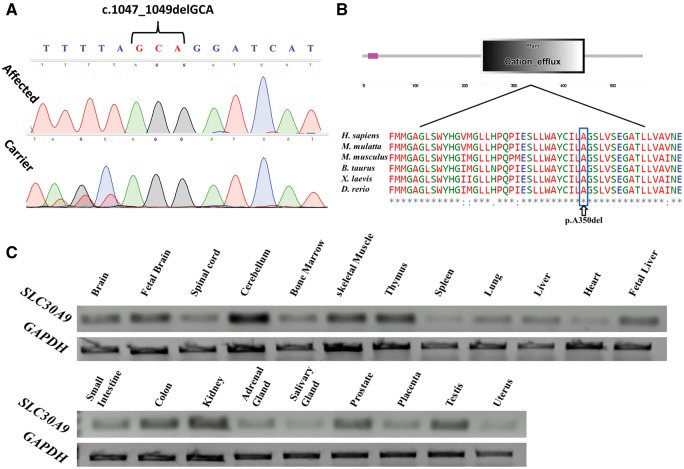
**The *SLC30A9* mutation, conservation and expression pattern.** (**A**) Sanger sequencing of an affected individual (Patient V:12) and obligatory carrier (Subject IV:3). (**B**) Above is the predicted domain architecture of SLC30A9 showing its cation efflux domain. Below is a multiple sequence alignment of selected *SLC30A9* orthologues. The in-frame deletion mutation is predicted to cause a p.(A350del) (marked with arrow) in a highly conserved residue within the putative cation efflux domain of SLC30A9 (boxed in blue). (**C**) RT-PCR of 21 normal human tissues demonstrating the expression pattern of *SLC30A9*.

Whole-exome sequencing data of Patients V:9 and V:15 (Fig. 1A) (complemented with Sanger sequencing of exons not covered) were filtered for normal variants as described in the ‘Materials and methods’ section. Following the above filtering, only a single homozygous variant was found within the 18 Mb locus: c.1047_1049delGCA, p.(A350del) in *SLC30A9* (termed also ZnT-9), within the cation efflux domain of the mature encoded protein (Fig. 2A and B). The *SLC30A9* mutation, validated by Sanger sequencing, was found to segregate within the family as expected for autosomal recessive heredity. The mutation has not been previously reported in the dbSNP database (http://www.ncbi.nlm.nih.gov/projects/SNP/) the 1000 Genomes project, (http://browser.1000genomes.org/index.html), the NHLBI Exome Sequencing Project (ESP; http://evs.gs.washington.edu/EVS/) or in the Exome Aggregation Consortium (ExAC) Browser (http://exac.broadinstitute.org/). *SLC30A9* encodes a 568 amino acid protein that is highly conserved throughout evolution (Fig. 2B). Screening of the mutation in 300 ethnically matched controls (600 chromosomes) identified a single carrier and no homozygous mutants (data not shown). Only 11 SLC30A9 loss-of-function mutations (stop gain, frameshift or essential splice site mutations) have been reported in the Exome Aggregation Consortium (ExAC), none of which are in a homozygous state.

### SLC30A9 expression patterns and subcellular localization

**Figure 3:**
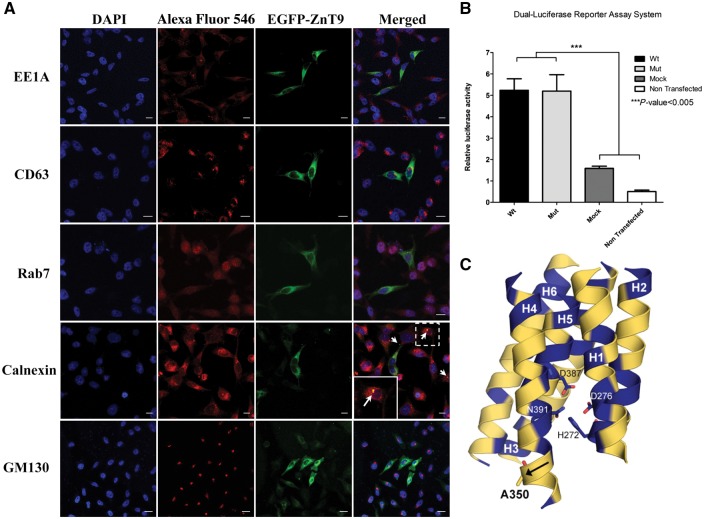
**SLC30A9 protein subcellular localization, effect on Wnt signaling and structural modeling.** (**A**) Overexpression of SLC30A9 protein fused to enhanced green fluorescent protein (EGFP) and immunofluorescent staining of different endosomal, Golgi and ER markers (CD63, EEA1, Rab7, GM130 and Calnexin) showing partial co-localization with endoplasmic reticulum in SH-SY5Y cells. On the *bottom left* side of the merged image of Calnexin marker panel is a magnification of the dashed frame, showing the co-localization in a single cell. Scale bars = 10 µm. (**B**) TOP flash reporter assay of neuroblastoma (SH-SY5Y) cells showing enhanced Wnt signalling in both SLC30A9 wild-type and mutant transfected cells. There was no significant difference between wild-type and mutant transfected cells. (**C**) Structural model of ZnT-9 protein showing the p.(A350del) in the fourth transmembrane helix, putatively causing destabilization of the protein structure in general, affecting the TM metal binding site specifically.

Analysis of *SLC30A9* expression in various normal human tissues by reverse transcriptase polymerase chain reaction (RT-PCR) demonstrated that it is ubiquitous. However, while *SLC30A9* is moderately expressed in most tissues examined, it is highly transcribed in foetal brain, cerebellum, skeletal muscle and kidney (Fig. 2C). Confocal analysis of the subcellular localization of SLC30A9 in SH-SY5Y cells transfected with expression vectors (pEGFP-C3) of a wild-type and mutant SLC30A9 fused to EGFP at its N-terminus were analysed (Fig. 3A). No difference in localization was observed for wild-type versus mutant proteins (not shown). Initial localization studies demonstrated cytoplasmic vesicles, possibly endosomes. Further in-depth subcellular co-localization studies with specific endosomal and Golgi markers (EEA1, Rab7, CD63 and GM130) failed to demonstrate co-localization with any of the markers examined. Staining of cells transfected with SLC30A9 fused to EGFP at its N-terminal with antibodies to calnexin (a widely used endoplasmic reticulum marker), demonstrated some co-localization of both, though this was only partial (Fig. 3A). Repeating these experiments with SLC30A9 fused to EGFP at its C termius (pEGFP-N2 constructs) yielded very low or absent fluorescence (not shown).

### Protein structural predictions and functional analysis

Previous publications have established a role of SLC30A9 in Wnt signalling (Chen *et al.*, 2005, 2007), which is involved in multiple developmental processes. To test possible effects of the mutation on this activity of SLC30A9, we performed a dual-luciferase reporter assay in neuroblastoma (SH-SY5Y) cells transfected with either wild-type or mutant SLC30A9-overexpressing constructs [pCDNA^TM^ 3.1 (−) under control of the CMV promoter]. In line with previously published data, enhanced Wnt signalling was seen in SLC30A9-transfected cells; however, there was no significant difference between the wild-type and mutant transfected cells (Fig. 3B).

**Figure 4:**
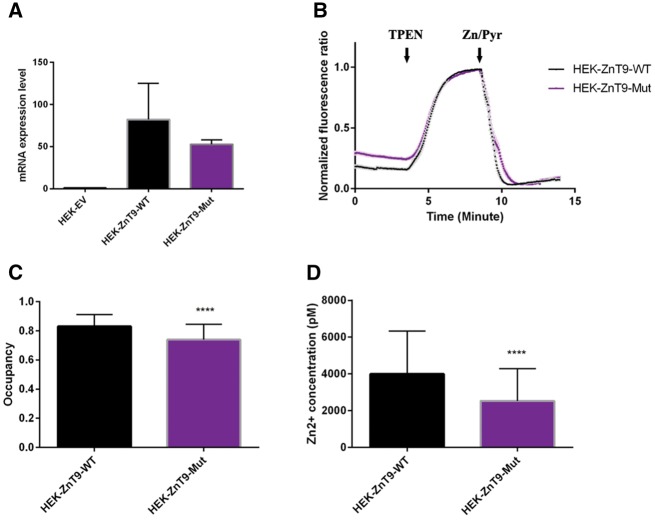
**Cytosolic free zinc measurements of HEK293 cells overexpressing wild-type or mutant ZnT-9.** (**A**) ZnT9 expression was assessed by quantitative RT-PCR and expression levels were equivalent in ZnT9-WT and ZnT9-mut expressing cells. (**B**) Normalized average traces obtained from three independent experiments (*n* = 90 cells for HEK-ZnT9-WT and *n* = 119 cells for HEK-ZnT9-mut). Steady-state fluorescence intensity ratio (citrine to cerulean) was first measured before obtaining the R_max_ under perfusion with KHB buffer containing the zinc chelator N,N,N’,N’-tetrakis (2-pyridylmethyl) ethylenediamine (50 μM; zinc-free condition). Finally, the R_min_ was obtained under perfusion with KHB buffer containing 5 μM pyrithione and 100 μM Zn^2+^ (zinc-saturated condition), providing saturating intracellular Zn^2+^ concentrations. (**C**) The probe occupancy was determined for ZnT-9 wild-type and mutant expressing cells (**D**) free zinc concentration was calculated using the formula: [Zn^2+^] = Kd (R_max_ − R)/(R − R_min_), revealing significant decreases in cytosolic free zinc levels in cells expressing the mutant form compare to cells expressing wild-type ZnT-9 (^****^P-value < 0.0001).

The p.(A350del) mutation is at the highly conserved putative cation-efflux domain of the mature encoded SLC30A9 protein. To assess how the mutation might affect SLC30A9/ZnT-9 function we generated a structural model for ZnT-9. Our model showed that p.(A350del) is in the fourth helix of the transmembrane domain (TM H4; Fig. 3C) and faces the membrane. Upon deletion of alanine at amino acid position 350, two scenarios are probable: (i) disorientations of all the C-terminal residues in H4 (100° turn, see direction in Fig. 3C); and (ii) disorientations of the three N-terminal residues of A350 in H4. Both cases can lead to: (i) a shorter helix formation, which will increase H4 tension and deform its structure, as the membrane dimensions are suitable to a longer helix; and (ii) insertion of residues from a loop to force a helix with proper length. In any of these cases, polar interactions and salt bridge interruption, distortion of the hydrophobic interactions between the protein residues and between residues to membrane lipids, are likely to take place. This is expected to cause destabilization of the protein structure in general and the relatively close transmembrane metal binding site specifically (Fig. 3C), putatively culminating in defective function. Due to this predicted deleterious effect, we hypothesized that a dysfunction of the putative cation efflux domain of ZnT-9 will lead to deficient Zn^2+^ transport, which will in turn give rise to differences in cytosolic free zinc in mutant cells. We therefore used the eCALWY family of genetically encoded Foürster Resonance Energy Transfer (FRET) Zn^2+^ probes (Vinkenborg *et al.*, 2009; Chabosseau *et al.*, 2014) to allow measurements of free cytosolic zinc in SH-SY5Y-transfected cells overexpressing either wild-type or mutant ZnT-9. Overexpression of the construct in transfected cells was assessed by quantitative RT-PCR and revealed similar levels in wild-type and mutant ZnT-9-expressing cells (Fig. 4A). Normalized average traces obtained from three independent experiments and the steady-state fluorescence intensity ratio (citrine to cerulean) was first measured before obtaining the R_max_ under perifusion with KHB buffer containing the zinc chelator N,N,N’,N’-tetrakis (2-pyridylmethyl) ethylenediamine (50 μM; zinc-free condition). Finally, the R_min_ was obtained under perifusion with KHB buffer containing 5 μM pyrithione and 100 μM Zn^2+^ (zinc-saturated condition), providing saturating intracellular Zn^2+^ concentrations (Fig. 4B). The probe occupancy was determined for both wild-type and mutant ZnT-9 expressing cells (Fig. 4C) and the free cytosolic zinc concentration was calculated. Interestingly, there was a significant decrease in cytosolic free zinc levels in cells expressing the mutant form compared to cells expressing wild-type ZnT-9 (Fig. 4B).

## Discussion

The kindred studied, similar to many of the Bedouin community in southern Israel, originates from Saudi Arabia. This community is unique in its high consanguinity and extreme inbreeding within clans, with high incidence of monogenic diseases (Markus *et al.*, 2013). Disease carrier rates range from 1:150 for diseases such as hypotonia, infantile, with psychomotor retardation and characteristic facies 2 (UNC80, MIM: #616801) (Perez *et al.*, 2015) to as high as 1:10 in some tribes for mitochondrial complex III deficiency, nuclear type 4 (UQCRQ, MIM: #615159) (Barel *et al.*, 2008). Here we describe a novel autosomal recessive cerebro-renal syndrome, with non-structural multifunctional CNS involvement and a renal phenotype that somewhat resembles that of ciliopathies. Homozygosity mapping identified a single 18 Mb disease-associated locus on chromosome 4 (LOD score 4.4 at D4S2971) shared by all affected individuals (Fig. 1B). Sequencing ruled out mutations in *WDR19* (NPHP13, MIM: #608151) within this locus or in any of the other known NPHP genes. Exome sequencing revealed a novel *SLC30A9* c.1047_1049delGCA, p.(A350del) mutation as the only likely damaging variant within the locus (Fig. 2A and B). The mutation segregated within the kindred as expected for recessive heredity and was not found in a homozygous state in 300 ethnically matched controls.

We demonstrated that *SLC30A9* is highly expressed in the human brain, skeletal muscle and kidney (Fig. 2C). SLC30A9 (ZnT-9) is a highly conserved protein that belongs to the solute carrier family 30 (SLC30) of zinc (Zn^2+^) transporters. Zn is an essential trace element and is widely required in various cellular functions. Intracellular Zn homeostasis is strictly regulated by Zn binding proteins and Zn transporters. Thus, dysfunction of such transport is likely to perturb Zn homeostasis. Indeed, many health problems such as growth retardation, immunodeficiency, hypogonadism, and neuronal and sensory dysfunctions have been associated with Zn homeostasis. Zn has a major role as a signalling mediator and acts as an intracellular signalling molecule, capable of communicating between cells, converting extracellular stimuli to intracellular signals, and controlling intracellular events (Fukada *et al.*, 2011). Importantly, Zn also plays a significant role in brain development and in proper brain function at every stage of life (Tyszka-Czochara *et al.*, 2014). Specifically, alterations in brain Zn status have been implicated in a wide range of neurological disorders including disorders of impaired brain development and of neurodegeneration (Prakash *et al.*, 2015). In addition, Zn is highly associated with chronic inflammatory diseases. For instance, many studies have shown high prevalence of Zn deficiency in chronic kidney disease (CKD) patients (Lobo *et al.*, 2010). Furthermore, intracellular Zn has an effect on translocation of NF-κB, a transcription factor critical for the expression of pro-inflammatory cytokines (Vasto *et al.*, 2006). However, the molecular mechanisms by which Zn contributes to inflammatory diseases have yet to be fully understood.

It is noteworthy that four of six affected individuals presented also with a renal phenotype of early-onset nephropathy with features of tubulo-interstitial nephritis, hypertension and tendency for hyperkalemia, as described for NPHP2 (inversin) mutations (Haider *et al.*, 1998). The origins of such hyperkalaemia, which appears early in the course of chronic kidney disease, are yet to be determined but might possibly reflect resistance to the kaliuretic effects seen in pseudohypoaldosteronism, as described for some forms of NPHP (Eisenstein *et al.*, 1992).

*NPHP13* resides within the disease-associated genomic locus; however, the affected individuals had no mutations in this gene. Moreover, unlike the classical clinical NPHP-associated phenotypes, none of the patients had renal cysts or rapid deterioration of renal function, liver fibrosis, cardiac malformations, or ciloapathy-related phenotypic features such as situs inversus or the molar tooth sign seen in MRI in Joubert syndrome and some cases of NPHP (Hildebrandt and Zhou, 2007; Halbritter *et al.*, 2013).

To date, mutations in two of the SLC30 gene family members have been identified as a cause of monogenic human diseases: a dominant form of neonatal Zn deficiency (TNZD, MIM: #608118), which results from reduced Zn in maternal breast milk, caused by maternal heterozygous SLC30A2 mutation (Chowanadisai *et al.*, 2006) and an autosomal recessive hypermanganesaemia (HMNDYT1, MIM: #613280), a metabolic disorder characterized by increased serum manganese, motor neurodegeneration with extrapyramidal features, polycythaemia, and hepatic dysfunction caused by homozygous mutations in SLC30A10 (Quadri *et al.*, 2012). Moreover, there have been previous reports showing association between SLC30 genes to different diseases: an R325W variant of ZnT8 was shown to be associated with type-2 diabetes by a genome-wide association study (Sladek *et al.*, 2007; Nicolson *et al.*, 2009). Also, increased *SLC30A6* (ZnT6) mRNA levels were detected in post-mortem cortex of patients with Alzheimer’s disease (Beyer *et al.*, 2012). Interestingly, different homozygous knock-out mice models (viewed in Mouse Genome Informatics) of the SLC30 Zn^2+^ transporters have been shown to cause severe abnormalities including growth retardation, dermatitis, reduced circulating insulin levels, skeletal defects and even embryonic lethality (http://www.informatics.jax.org/).

The function of SLC30A9 is not fully understood. Previous studies suggested nuclear receptor co-activation in a hormone-dependent and GRIP1-dependent manner (Chen *et al.*, 2005), and activation of Wnt signalling through β-catenin interaction (Chen *et al.*, 2007). However, our assay in SH-SY5Y cells overexpressing mutant and wild-type SLC30A9 showed that while SLC30A9 indeed affects Wnt signalling, this function of SLC30A9 was not affected by the disease-causing mutation. Thus, the mechanism by which the mutation causes the phenotype is probably irrelevant to the Wnt signalling pathway (Fig. 3B).

To better understand the molecular mechanism of the disease, we set out to determine the subcellular localization of SLC30A9 via confocal analysis of SH-SY5Y cells overexpressing wild-type versus mutant SLC30A9 fused to GFP. There were no differences in the localization of the wild-type and mutant proteins, implying that the mutation does not affect localization of the protein, but, rather, its function. Interestingly, both wild-type and mutant SLC30A9 were not seen in the nucleus. Rather, they were expressed within vesicular cytosolic compartments likely of the endoplasmic reticulum but did not co-localize with various endosomal and Golgi markers (Fig. 3A).

Per Clustal Omega analysis (http://www.ebi.ac.uk/Tools/msa/clustalo/) (Sievers *et al.*, 2011), the *SLC30A9* deletion mutation occurs at an alanine residue in a highly conserved cation efflux domain (Fig. 2B). This would presumably have a structural effect on a hydrophobic alpha helix transmembrane motif within this domain. Indeed, our protein model shows that the p.A350 deletion is likely to cause distortion of the hydrophobic interactions between the residues, and between the residues and membrane lipids. This will ultimately lead to destabilization of the protein structure and will probably cause defective function. In terms of protein structure, ZnT-9 is part of the cation diffusion facilitator (CDF) protein family, though it is unique among CDF proteins. This is supported by multiple sequence alignment between ZnT-9 sequence and other human ZnT (hZnT) proteins, which showed that ZnT-9 transmembrane helices do not contain many of the conserved residues as all other ZnT proteins, according to the phylogenetic tree. Hence, we modelled the transmembrane domain (which contains A350) of ZnT-9 manually, based on different CDF proteins’ multiple sequence alignments and YiiP structure (Lu and Fu, 2007). We performed 20 independent multiple sequence alignments that showed that helices 1, 2, 3 and 6 are conserved in all multiple sequence alignments, while H4 is less conserved and H5 varies between different multiple sequence alignments. The final model together with multiple sequence alignment of hZnT proteins suggest that the transmembrane metal binding site, which is composed of two residues from H2 and two from H5, which were shown in many studies to be related with the CDFs metal specificity (Kolaj-Robin *et al.*, 2015), contains an HD-DN signature. Other ZnT proteins contain an HD-HD motif or ND-HD signature, which were shown to be related with Zn and Mn specificity, respectively (Hoch *et al.*, 2012; Nishito *et al.*, 2016). This unique signature of ZnT-9 can imply different metal specificities other than Zn, and a possible role in transition metal homeostasis. Nevertheless, measurements of free cytosolic Zn^2+^ in SH-SY5Y cells overexpressing SLC30A9 showed a significant decrease in cytosolic free Zn^2+^ levels in cells expressing the mutant form compared to cells expressing wild-type ZnT-9. This might be explained by several options: first, the mutation could be hypermorphic, causing gain of function, though considering the nature of the mutation in context of our protein model predictions this is unlikely. Second, in our experimental conditions, ZnT-9 transporter might be operating in the reverse direction, allowing Zn^2+^ into the cytosol, wherein the mutant is less active. Either way, our experimental data imply that ZnT-9 plays a role in intracellular Zn^2+^ transport. Altogether, our data demonstrate that this novel syndrome of intellectual disability, muscle weakness and oculomotor apraxia with partially penetrant early onset nephropathy is caused by an *SLC30A9* mutation, likely through abnormal intracellular Zn homeostasis rather than through Wnt signalling disruption. Furthermore, we suggest a dual function for the orphan ZnT-9 transporter: aside from its previously demonstrated role in transcriptional activation of Wnt signalling, it acts also in maintaining intracellular Zn homeostasis.

## Web resources

UCSC genome browser: https://genome.ucsc.edu/

Chromas: http://technelysium.com.au/

HaploPainter: http://haplopainter.sourceforge.net/index.html

Primer3 (V.0.4.0): http://frodo.wi.mit.edu/primer3/

SNP database: http://www.ncbi.nlm.nih.gov/projects/SNP/

Clustal Omega software: http://www.ebi.ac.uk/Tools/msa/clustalo/

NHLBI Exome Sequencing Project, Seattle, WA: http://evs.gs.washington.edu/EVS/

Marshfield Maps: http://research.marshfieldclinic.org/genetics/

Online Mendelian Inheritance in Man (OMIM): http://www.omim.org/

Biotechnology Information GenBank: http://www.ncbi.nlm.nih.gov/

HomozygosityMapper: http://www.homozygositymapper.org/

Superlink-Online SNP: http://cbl-hap.cs.technion.ac.il/superlink-snp/

1000 genomes project: http://www.1000genomes.org/

PolyPhen-2: http://genetics.bwh.harvard.edu/pph2/

ExAC Browse: http://exac.broadinstitute.org/

Mouse Genome Informatics: http://www.informatics.jax.org/

## Abbreviations

EGFP: enhanced green fluorescent protein
NPHP: nephronophthisis

## References

[awx013-B1] Badhwar A, Berkovic SF, Dowling JP, Gonzales M, Narayanan S, Brodtmann A Action myoclonus-renal failure syndrome: Characterization of a unique cerebro-renal disorder. Brain 2004; 127: 2173–82. 15364701 10.1093/brain/awh263

[awx013-B2] Barel O, Shorer Z, Flusser H, Ofir R, Narkis G, Finer G Mitochondrial complex III deficiency associated with a homozygous mutation in UQCRQ. Am J Hum Genet 2008; 82: 1211–6. 18439546 10.1016/j.ajhg.2008.03.020 PMC2427202

[awx013-B3] Beyer N, Coulson DTR, Heggarty S, Ravid R, Hellemans J, Irvine GB Zinc transporter mRNA levels in alzheimer’s disease postmortem brain. J Alzheimer’s Dis 2012; 29: 863–73. 22349685 10.3233/JAD-2012-112105

[awx013-B4] Bockenhauer D, Feather S, Stanescu HC, Bandulik S, Zdebik AA, Reichold M Epilepsy, Ataxia, Sensorineural Deafness, Tubulopathy, and KCNJ10 Mutations. N Engl J Med 2009; 360: 1960–70. 19420365 10.1056/NEJMoa0810276 PMC3398803

[awx013-B5] Bökenkamp A, Ludwig M The oculocerebrorenal syndrome of Lowe: an update. Pediatr Nephrol 2016: 1–12. 10.1007/s00467-016-3343-3 PMC5118406 27011217

[awx013-B6] Chabosseau P, Tuncay E, Meur G, Bellomo EA, Hessels A, Hughes S Mitochondrial and ER-targeted eCALWY probes reveal high levels of free Zn2+. ACS Chem Biol 2014; 9: 2111–20. 25011072 10.1021/cb5004064 PMC6101202

[awx013-B7] Chen Y-H, Kim JH, Stallcup MR GAC63, a GRIP1-dependent nuclear receptor coactivator. Mol Cell Biol 2005; 25: 5965–72. 15988012 10.1128/MCB.25.14.5965-5972.2005 PMC1168828

[awx013-B8] Chen YH, Yang CK, Xia M, Ou CY, Stallcup MR Role of GAC63 in transcriptional activation mediated by beta-catenin. Nucleic Acids Res 2007; 35: 2084–92. 17344318 10.1093/nar/gkm095 PMC1874623

[awx013-B9] Chowanadisai W, Lönnerdal B, Kelleher SL Identification of a mutation in SLC30A2 (ZnT-2) in women with low milk zinc concentration that results in transient neonatal zinc deficiency. J Biol Chem 2006; 281: 39699–707. 17065149 10.1074/jbc.M605821200

[awx013-B10] Cubillas C, Vinuesa P, Tabche ML, García-de los Santos A Phylogenomic analysis of Cation Diffusion Facilitator proteins uncovers Ni2+/Co2+ transporters. Metallomics 2013; 5: 1634–43. 24077251 10.1039/c3mt00204g

[awx013-B11] Eisenstein B, Davidovitz M, Garty BZ, Shmueli D, Ussim A, Stark H Severe tubular resistance to aldosterone in a child with familial juvenile nephronophthisis. Pediatr Nephrol 1992; 6: 57–9. 1536742 10.1007/BF00856835

[awx013-B12] Emsley P, Cowtan K Coot: Model-building tools for molecular graphics. Acta Crystallogr Sect D Biol Crystallogr 2004; 60: 2126–32. 15572765 10.1107/S0907444904019158

[awx013-B13] Fukada T, Yamasaki S, Nishida K, Murakami M, Hirano T Zinc homeostasis and signaling in health and diseases. J Biol Inorg Chem 2011; 16: 1123–34. 21660546 10.1007/s00775-011-0797-4 PMC3176402

[awx013-B14] Guex N, Peitsch MC SWISS-MODEL and the Swiss-PdbViewer: an environment for comparative protein modeling. Electrophoresis 1997; 18: 2714–23. 9504803 10.1002/elps.1150181505

[awx013-B15] Haider NB, Carmi R, Shalev H, Sheffield VC, Landau D A Bedouin kindred with infantile nephronophthisis demonstrates linkage to chromosome 9 by homozygosity mapping. Am J Hum Genet 1998; 63: 1404–10. 9792867 10.1086/302108 PMC1377550

[awx013-B16] Halbritter J, Porath JD, Diaz KA, Braun DA, Kohl S, Chaki M Identification of 99 novel mutations in a worldwide cohort of 1,056 patients with a nephronophthisis-related ciliopathy. Hum Genet 2013; 132: 865–84. 23559409 10.1007/s00439-013-1297-0 PMC4643834

[awx013-B17] Hall AM, Unwin RJ, Hanna MG, Duchen MR Renal function and mitochondrial cytopathy (MC): More questions than answers? Qjm 2008; 101: 755–66. 18487272 10.1093/qjmed/hcn060

[awx013-B18] Hildebrandt F, Zhou W Nephronophthisis-associated ciliopathies. J Am Soc Nephrol 2007; 18: 1855–71. 17513324 10.1681/ASN.2006121344

[awx013-B19] Hoch E, Lin W, Chai J, Hershfinkel M, Fu D, Sekler I Histidine pairing at the metal transport site of mammalian ZnT transporters controls Zn2+ over Cd2+ selectivity. Proc Natl Acad Sci U S A 2012; 109: 7202–7. 22529353 10.1073/pnas.1200362109 PMC3358884

[awx013-B20] Kolaj-Robin O, Russell D, Hayes KA, Pembroke JT, Soulimane T Cation Diffusion Facilitator family: Structure and function. FEBS Lett 2015; 589: 1283–95. 25896018 10.1016/j.febslet.2015.04.007

[awx013-B21] Lobo JC, Torres JPM, Fouque D, Mafra D Zinc deficiency in chronic kidney disease: is there a relationship with adipose tissue and atherosclerosis?. Biol Trace Elem Res 2010; 135: 16–21. 19760368 10.1007/s12011-009-8504-9

[awx013-B22] Lu M, Fu D Structure of the zinc transporter YiiP. Science 2007; 317: 1746–8. 17717154 10.1126/science.1143748

[awx013-B23] Markus B, Alshafee I, Birk OS Deciphering the fine-structure of tribal admixture in the Bedouin population using genomic data. Heredity (Edinb) 2013; 112: 182–9. 24084643 10.1038/hdy.2013.90 PMC3907104

[awx013-B24] Nicolson TJ, Bellomo EA, Wijesekara N, Loder MK, Baldwin JM, Gyulkhandanyan AV Insulin storage and glucose homeostasis in mice null for the granule zinc transporter ZnT8 and studies of the type 2 diabetes-associated variants. Diabetes 2009; 58: 2070–83. 19542200 10.2337/db09-0551 PMC2731533

[awx013-B25] Nishito Y, Tsuji N, Fujishiro H, Takeda T, Yamazaki T, Teranishi F Direct comparison of manganese detoxification/efflux proteins and molecular characterization of znt10 protein as a manganese transporter. J Biol Chem 2016; 291: 14773–87. 27226609 10.1074/jbc.M116.728014 PMC4938194

[awx013-B26] Perez Y, Kadir R, Volodarsky M, Noyman I, Flusser H, Shorer Z UNC80 mutation causes a syndrome of hypotonia, severe intellectual disability, dyskinesia and dysmorphism, similar to that caused by mutations in its interacting cation channel NALCN. J Med Genet 2016; 53: 397–402. 26545877 10.1136/jmedgenet-2015-103352

[awx013-B27] Prakash A, Bharti K, Majeed ABA Zinc: Indications in brain disorders. Fundam Clin Pharmacol 2015; 29: 131–49. 25659970 10.1111/fcp.12110

[awx013-B28] Quadri M, Federico A, Zhao T, Breedveld GJ, Battisti C, Delnooz C Mutations in SLC30A10 cause parkinsonism and dystonia with hypermanganesemia, polycythemia, and chronic liver disease. Am J Hum Genet 2012; 90: 467–77. 22341971 10.1016/j.ajhg.2012.01.017 PMC3309204

[awx013-B29] Seelow D, Schuelke M, Hildebrandt F, Nürnberg P HomozygosityMapper - An interactive approach to homozygosity mapping. Nucleic Acids Res 2009; 37: 593–9. 10.1093/nar/gkp369 PMC2703915 19465395

[awx013-B30] Sievers F, Wilm A, Dineen D, Gibson TJ, Karplus K, Li W Fast, scalable generation of high-quality protein multiple sequence alignments using Clustal Omega. Mol Syst Biol 2011; 7: 539. 21988835 10.1038/msb.2011.75 PMC3261699

[awx013-B31] Silberstein M, Tzemach A, Dovgolevsky N, Fishelson M, Schuster A, Geiger D Online system for faster multipoint linkage analysis via parallel execution on thousands of personal computers. Am J Hum Genet 2006; 78: 922–35. 16685644 10.1086/504158 PMC1474109

[awx013-B32] Sladek R, Rocheleau G, Rung J, Dina C, Shen L, Serre D A genome-wide association study identifies novel risk loci for type 2 diabetes. Nature 2007; 445: 881–5. 17293876 10.1038/nature05616

[awx013-B33] Tyszka-Czochara M, Grzywacz A, Gdula-Argasińska J, Librowski T, Wiliński B, Opoka W The role of zinc in the pathogenesis and treatment of central nervous system (CNS) diseases. Implications of zinc homeostasis for proper CNS function. Acta Pol Pharm 2014; 71: 369–77. 25265815

[awx013-B34] Vasto S, Mocchegiani E, Candore G, Listì F, Colonna-Romano G, Lio D Inflammation, genes and zinc in ageing and age-related diseases. Biogerontology 2006; 7: 315–27. 16972155 10.1007/s10522-006-9046-6

[awx013-B35] Vinkenborg JL, Nicolson TJ, Bellomo EA, Koay MS, Rutter GA, Merkx M Genetically encoded FRET sensors to monitor intracellular Zn2+ homeostasis. Nat Meth 2009; 6: 737–40. 10.1038/nmeth.1368 PMC6101214 19718032

[awx013-B36] Wolf MTF Nephronophthisis and related syndromes. Curr Opin Pediatr 2015; 27: 201–11. 25635582 10.1097/MOP.0000000000000194 PMC4422489

[awx013-B37] The PyMOL Molecular Graphics System, Version 1.7.4 Schrödinger, LLC. 2014

